# Supplementary material for: Thirteen Ovary-Enriched Genes Are Individually Not Essential for Female Fertility in Mice
Source: Cells. 2024 May 8;13(10):802. doi: 10.3390/cells13100802 (PMC11119756; doi:10.3390/cells13100802)
Supplement: Supplementary file 1 [file cells-13-00802-s001.zip › Pham et al., Supplementary Figures_Revised.pdf]

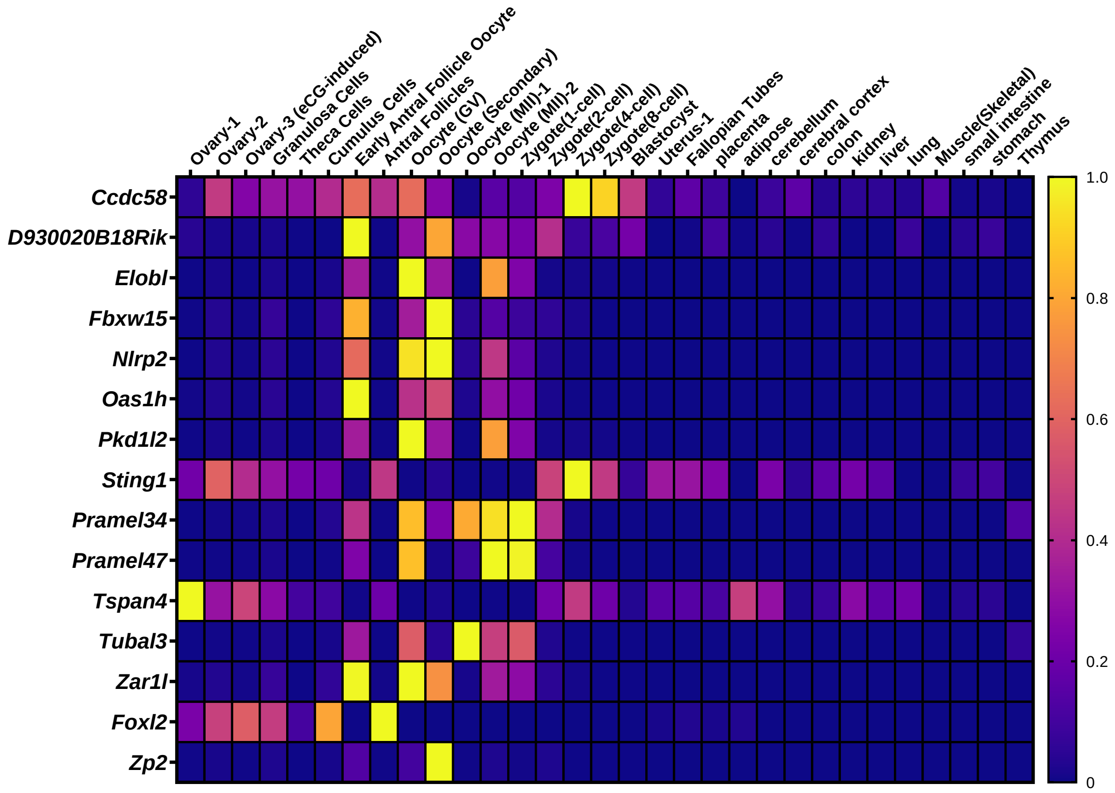

Figure S1. Expression patterns of candidate genes in mice, using the TPM values obtained from Mammalian Reproductive Genetics database v2.0, the expression levels of 13 genes (*Ccdc58*, *D930020B18Rik*, *Elobl*, *Fbxw15*, *Nlrp2*, *Oas1h*, *Pkd1l2*, *Pramel34*, *Pramel47*, *Sting1*, *Tspan4*, *Tubal3*, *Zar1l*) were shown as heatmap. The expression patterns of *Foxl2* and *Zp2*, which are well known to have important role in the ovary, are also shown at the bottom).

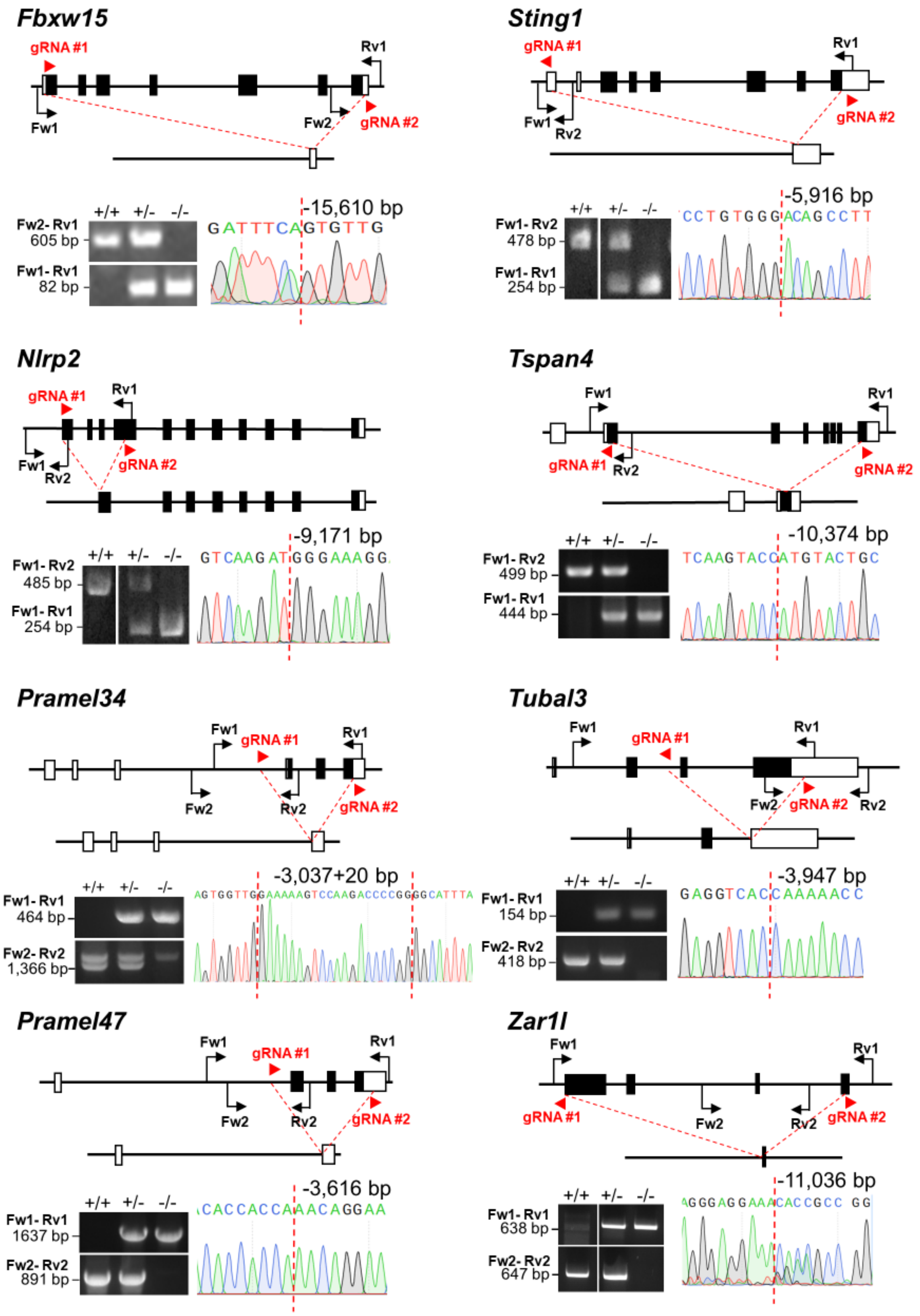

Figure S2. KO strategy, genotyping PCR and Sanger sequencing of *Fbxw15*, *Nlrp2*, *Pramel34*, *Pramel47*, *Sting1*, *Tspan4*, *Tubal3*, and *Zar1l*. Fw1/2: forward primer for the genotyping. Rv1/2: Reverse primers for the genotyping.
