# Supplementary material for: Thirteen Ovary-Enriched Genes Are Individually Not Essential for Female Fertility in Mice
Source: Cells. 2024 May 8;13(10):802. doi: 10.3390/cells13100802 (PMC11119756; doi:10.3390/cells13100802)
Supplement: Supplementary file 1 [file cells-13-00802-s001.zip › Pham et al.,Supplementary Tables.pdf]

| **Gene symbol** | gRNA sequences | | PAM |
|---|---|---|---|
| *Ccdc58* | U | ACGTTATTTATGTTGCCGAG | TGG |
| | D | ACTCGGTCATCTCATCGCCT | GGG |
| *D930020B18Rik* | U | GCCATGCGACACCCGCGACC | AGG |
| | D | TGTCCCCACGCCCAGTTCTG | GGG |
| *Elobl* | U | AGCCATTCAGTTATACCCAG | GGG |
| | D | CCCTAATCAATTTTTTGATG | AGG |
| *Fbxw15* | U | TCATCGGCAAACAAGGTAAA | TGG |
| | D | AAAGGCCTATTTGCAAGCCC | AGG |
| *Nlrp2* | U | AGTCACCGTCCAGATCTTAG | AGG |
| | D | GTGATTCGAGATCTAATCCT | GGG |
| *Oas1h* | U | GAGCAATCCGAGTGCTGCTA | AGG |
| | D | AATCTGTCTAGTGATCATCG | AGG |
| *Pkd1l2* | U | TGGTGGATCGGACTCACGGG | AGG |
| | D | CAGCTGCAGTTATATCCGGG | AGG |
| *Pramel34* | U | AAGTGGTTGATGTGATGGTG | AGG |
| | D | GTGGGGTCAGAAAAACAGTG | TGG |
| *Pramel47* | U | ACCTCTTCATCCTAAACTGG | GGG |
| | D | GCTTTAATGAGTTTACTAAC | AGG |
| *Sting1* | U | CAAAAGTGACAGGGCCCCAC | AGG |
| | D | ACTGACCTCATCTGAGGCAT | GGG |
| *Tspan4* | U | GCAGGTTGAAGGCGAACATG | AGG |
| | D | ACATGGTCATAGCGAAGGTG | AGG |
| *Tubal3* | U | CTGGTTGGCGTTGGTAGGCA | AGG |
| | D | TTATATATCTAATGAATTAG | AGG |
| *Zar1l* | U | CAGAAAAGACGCTCCATTTG | AGG |
| | D | AAAGACACATTGATGTCCGG | AGG |

**Table S1.** The sequences of gRNAs used for generating 13 different knockout mouse lines (*Ccdc58, D930020B18Rik, Elobl, Fbxw15, Nlrp2, Oas1h, Pkd1l2, Pramel34, Pramel47, Sting1, Tspan4, Tubal3, Zar1l*); U: Upstream of the target site, D: Downstream of the target site.



*Cells* **2024**, *13*, 802. https://doi.org/10.3390/cells13100802 www.mdpi.com/journal/cells

| Gene symbol | Alleles | Forward Primer | Reverse Primer | Annealing | Elongation | Band size (bp) |
|---|---|---|---|---|---|---|
| *Ccdc58* | KO | TTTGTGGGGAAAGGGGTACT | GATGGAACCTGTCCTCTGGA | 60°C, 30 s | 72°C, 30 s | 645 |
| | WT | TTTGTGGGGAAAGGGGTACT | CATACAGCTGTTTACAGGTTTGG | 60°C, 30 s | 72°C, 30 s | 1,025 |
| *D930020B18Rik* | KO | TCCGCCCATCGCTGTGTAGG | TCCAATCGCCAAGGAGACCC | 60°C, 30 s | 72°C, 30 s | 577 |
| | WT | AGATGGCGTTTGTGGCCAGG | TACACGTCTGTGCAACGTGG | 60°C, 30 s | 72°C, 30 s | 677 |
| *Elobl* | KO | CCAGGGCTGAGCAAAGTAAG | TGTAGCAGGCCCTATCATCC | 65°C, 30 s | 72°C, 30 s | 672 |
| | WT | TATTAATTGAATCCTTGGCTTTTGA | CTACCTGACTAGTGAAGCCACATTC | 65°C, 30 s | 72°C, 30 s | 876 |
| *Fbxw15* | KO | AGGCCACTGAAGATGGCGATCC | TGCACCATCACATTTGGCCTC | 60°C, 5 s | 68°C, 5 s | 82 |
| | WT | CATAGTCACATACCTACACACGCAC | TGCACCATCACATTTGGCCTC | 60°C, 5 s | 68°C, 5 s | 605 |
| *Nlrp2* | KO | GCTATGGCAGAAAGGTTACCAG | CATCTTCCTCTTCAGCAAACTG | 60°C, 5 s | 68°C, 5 s | 254 |
| | WT | GCTATGGCAGAAAGGTTACCAG | GGTGCTACCCATCATGCCTGAT | 60°C, 5 s | 68°C, 5 s | 485 |
| *Oas1h* | KO | ACAAACCCCAGAGTGTCAGG | CCTCCCCTTCACAGAGATTG | 60°C, 30 s | 72°C, 30 s | 726 |
| | WT | TTTGTCTGGTCAAGCACTGG | GTGACGGTGACCTGGTTTTT | 60°C, 30 s | 72°C, 30 s | 707 |
| *Pkd1l2* | KO | GCAGTGGTCCTAGGATGACC | TATGATCAGAGCATATTAGG | 60°C, 30 s | 72°C, 30 s | 685 |
| | WT | GCTTGTTCATGGACCTCTGATAC | GGTCTATTTTCACCAGCATTCAG | 60°C, 30 s | 72°C, 30 s | 669 |
| *Pramel34* | KO | AGCCATGCATATTCATTGGG | GTGTCTTTCCAATCCTGGACCT | 58°C, 30 s | 72°C, 30 s | 464 |
| | WT | GAGATAGGGTTTCTCTGTATA | TGTGTACTCCTCATGCTCCC | 58°C, 30 s | 72°C, 90 s | 1,366 |
| *Pramel47* | KO | ACCCACAGTTGTCATCCTCC | AGCAAAGGACTCATGTGCCA | 58°C, 30 s | 72°C, 90 s | 1,637 |
| | WT | TCCTGGTTTCAGCTCTGCAA | GCCATGACAGTCATAAAGCC | 58°C, 30 s | 72°C, 60 s | 891 |
| *Sting1* | KO | TGCTGTTCATCTCAATCTCTCCTG | CATAGTAGGGTTGGTTGAGGCTCAG | 60°C, 5 s | 68°C, 5 s | 254 |
| | WT | TGCTGTTCATCTCAATCTCTCCTG | AACAACTGCAACTCAGCTCGCTTC | 60°C, 5 s | 68°C, 5 s | 478 |
| *Tspan4* | KO | GCAGCAGCCTGTATGGGCGC | GTCTGGTGTCTGCTGCCACCC | 65°C, 30 s | 72°C, 30 s | 444 |
| | WT | GCAGCAGCCTGTATGGGCGC | GGTGAGGCAAGGACGTGGGC | 65°C, 30 s | 72°C, 30 s | 499 |
| *Tubal3* | KO | TATCCCATGGGTACATTGCCA | AGGCTCTCAGTTAGCAAAGGT | 58°C, 30 s | 72°C, 30 s | 154 |
| | WT | CACTGGTACATCACAGAAGG | TGCGTTGTTTTAGGGAAGGGG | 58°C, 30 s | 72°C, 30 s | 418 |
| *Zar1l* | KO | TCCACTACAGCACAGCCAAT | CCCCAAGGACTGAGTTACCA | 60°C, 30 s | 72°C, 30 s | 638 |
| | WT | AGAGGAGATGGGAGAGTGCTT | CACATGGTTCAGGGTCAAGA | 60°C, 30 s | 72°C, 30 s | 647 |

**Table S2.** The sequences of primes and PCR condition used for genotyping for 13 different knockout mouse lines (*Ccdc58, D930020B18Rik, Elobl, Fbxw15, Nlrp2, Oas1h, Pkd1l2, Pramel34, Pramel47, Sting1, Tspan4, Tubal3, Zar1l*).
